# “*Syndemic moral distress*”: sexual health provider practices in the context of co-occurring, socially produced sexual and mental health epidemics

**DOI:** 10.1186/s12913-022-08149-1

**Published:** 2022-06-06

**Authors:** Travis Salway, Stéphanie Black, Angel Kennedy, Sarah Watt, Olivier Ferlatte, Mark Gaspar, Rod Knight, Mark Gilbert

**Affiliations:** 1 Faculty of Health Sciences, Simon Fraser University, Blusson Hall, 8888 University Drive, Burnaby, BC V5A1S6 Canada; 2 BC Centre for Disease Control, 655 West 12th Avenue, Vancouver, BC V5Z4R4 Canada; 3 Centre for Gender and Sexual Health Equity, 1190 Hornby Street, 11th Floor, Vancouver, BC V6Z1Y6 Canada; 4 École de Santé Publique de L’Université de Montréal, 7101 Avenue du Parc, Montréal, Québec H3N1X9 Canada; 5 Centre de Recherche en Santé Publique, 7101 Avenue du Parc, Montréal, Québec H3N1X9 Canada; 6 Dalla Lana School of Public Health, University of Toronto, 155 College Street, Toronto, ON M5T3M7 Canada; 7 BC Centre On Substance Use, 1045 Howe Street, Suite 400, Vancouver, BC V6Z2A9 Canada; 8 Faculty of Medicine, University of British Columbia, 2194 Health Sciences Mall, Vancouver, BC V6T1Z3 Canada; 9 School of Population and Public Health, University of British Columbia, 2206 East Mall, BC V6T1Z3 Vancouver, Canada

**Keywords:** Sexual health, Syndemic theory, Moral distress, HIV, Mental health

## Abstract

**Background:**

‘Syndemic’ refers to socially produced, intertwined, and co-occurring epidemics. Syndemic theory is increasingly used to understand the population-level relationships between sexual health (including HIV) and mental health (including problematic substance use) epidemics. Syndemic-informed clinical interventions are rare.

**Methods:**

We therefore asked 22 sexual health practitioners from six sexual health clinics in British Columbia, Canada to define the word ‘syndemic’ and then asked how the theory related to their clinical practice.

**Results:**

Responses to syndemic theory ranged widely, with some practitioners providing nuanced and clinically informed definitions, others expressing a vague familiarity with the term, and others still having no prior knowledge of it. Where practitioners acknowledged the relevance of syndemic theory to their practice, they articulated specific ways in which syndemics create moral distress, that is, feeling that the most ethical course of action is different from what they are mandated to do. While some practitioners routinely used open-ended questions to understand the social and economic contexts of patients’ sexual health needs, they described an uneasiness at potentially having surfaced concerns that could not be addressed in the sexual health clinic. Many observed persistent social, mental health, and substance use-related needs among their patients, but were unable to find feasible solutions to these issues.

**Conclusions:**

We therefore propose that interventions are needed to support sexual health practitioners in addressing psychosocial health needs that extend beyond their scope of practice, thereby reducing ‘syndemic moral distress’.

## Background

Sexual health clinics are unique primary care settings that are funded and/or mandated to provide accessible and non-judgmental prevention and treatment services for HIV and other sexually transmitted and blood-borne infections (STBBI), as well as reproductive health care [1, 2]. Some socially definable groups—for example, sexual minority men (or gay, bisexual, and other men who have sex with men [GBMSM]) and residents of economically disadvantaged urban environments—experience elevated rates of STBBI [3, 4]. Consequently, these groups constitute a disproportionately large set of clients in urban sexual health clinics [5, 6]. In many cases, sexual minority men present at sexual health clinics experiencing high rates of co-occurring psychosocial and mental health concerns, including but not limited to anxiety, depression, harms associated with substance use, and partner violence [7, 8]. The population-level co-occurrence and interaction of multiple health outcomes has been termed a ‘syndemic’ (i.e., synergistic epidemic), because the sexual, psychosocial, and mental health issues are theorized to co-occur and reinforce one another and thereby exacerbate outcomes among marginalized populations [3]. Moreover, syndemics are understood as being socially and politically produced; many of the social conditions that disproportionately impact these groups (hereafter termed ‘syndemic-burdened populations’), including poverty, stigma, violence, and social exclusion, are fundamental causes to which syndemic burdens are attributable [3, 9].

Syndemic theory calls for new ways of doing health promotion and disease prevention work, by attending to broader health concerns of socially marginalized groups, in a holistic manner, and by addressing the root causes of ill health [3]. Yet, identifiable public health interventions to address syndemics are scarce, largely owing to what Singer describes as “funding sources [that] are outcomes-oriented, [and] disease programmes [that] continue to be vertical with unfounded prioritisation of some diseases” [3]. Within this funding scheme, public health responses to HIV and other STBBI epidemics have tended toward greater investment in biomedical models [10, 11]. In other words, a siloed approach to healthcare—in this case, related to STBBI prevention and treatment—has hampered efforts to treat co-occurring and synergistic health conditions holistically. Syndemic interventions that focus on supporting sexual health practitioners’ abilities to respond to these co-occurring conditions are even scarcer [2].

This focused STBBI mandate of sexual health clinics may in turn produce tension, conflict, or confusion for some providers, when they encounter co-occurring patient concerns which are beyond their scope of practice [2]. “Moral distress” is a cognitive and emotional response to conditions whereby a healthcare provider “knows the right thing to do, but institutional constraints make it nearly impossible to pursue the right course of action” [12]. While the phenomenon has primarily been described in the context of nursing, all healthcare providers are susceptible to moral distress insofar as they work in close relation with patients/clients^1^ who have needs that may be at odds with institutional and social expectations and norms [13]. In some healthcare settings where moral distress has been studied (e.g., critical care nursing), interventions have been proposed to alleviate moral distress, through interpersonal approaches like staff debriefing and counseling [13]. Approaches to address moral distress through structural changes—e.g., allocation of healthcare resources, changes to administrative policies and procedures, reorganization of services—have infrequently been explored [14, 15].

To our knowledge, no study has examined the knowledge and perspectives of syndemic theory and its relevance to clinicians directly working with syndemic-burdened populations. Furthermore, as syndemic research has become dominated by epidemiological methods, qualitative studies of syndemic are rare [16]. Qualitative research offers an opportunity for those engaged with syndemic thinking to move beyond the epidemiological research that describes syndemics to identify feasible and practical interventions to treat and prevent syndemics [16]. Therefore, in the present study, we draw on in-depth interviews with sexual health providers to investigate sexual health provider practices in response to syndemics. From these findings, we then define the particular phenomenon of ‘syndemic moral distress’ in the setting of low-barrier sexual health clinics. Reflecting on the results, we imagine individual-level interventions and structural reforms that may increase practitioners’ abilities to address the needs of syndemic-burdened populations, while decreasing their own feelings of moral distress.

## Methods

### Methodology

In this exploratory qualitative study, we used interpretive description to obtain sexual health provider perspectives and experiences related to syndemic theory. Thorne developed this methodology to recognize the unique ways in which healthcare providers gather and use knowledge [17]. It is an inductive approach, grounded in constructivist perspectives, that is particularly well-suited to generating clear understandings of complex experiential phenomena in applied settings, such as sexual health clinics.

### Sample

We purposively recruited 22 health professionals, including nurses (*n* = 16), physicians (*n* = 3), counselors (*n* = 2), and educators (*n* = 1) from six sexual health clinics in Greater Vancouver and Victoria, British Columbia (BC), Canada. The overarching goal of this study—as described elsewhere [2]—was to characterize sexual health providers’ perspectives with regard to addressing the mental health-related needs of their clients, including those related to substance use harm. Clinics were selected to capture diversity in funding models and client populations. Four of the clinics were public health-administered, meaning all staff were salaried employees or contractors paid from ‘global’ public health budgets, while the other two clinics were administered by non-profit organizations that rely on fee-for-service billings to the BC Medical Services Plan. All six clinics employ a mix of nurses and physicians, and one clinic additionally included counselors on staff. Four of the clinics were focused primarily on provision of STBBI testing and treatment, and the other two clinics primarily delivered reproductive health care (e.g., birth control, screening), as well as STBBI testing and treatment.

Participants were recruited through snowball sampling and word-of-mouth. We recruited participants iteratively over a 5-month period (March-July 2018), seeking participation from a diverse group of providers on the basis of geography (Vancouver being the largest urban center in BC, Victoria a smaller geographically distinct city), discipline (nurse, physician, other), years of practice, and clinic population (GBMSM, other). All participants provided written informed consent at the start of the interview, which typically lasted one hour. The study protocol was reviewed and approved by the University of British Columbia Research Ethics Board.

### Data collection

Individual in-depth interviews were conducted by the first two authors, both of whom were present for the first four interviews to ensure consistency in interview style and content. The interview followed a semi-structured guide that included four parts: (1) the participant’ role and experiences in sexual health clinical services; (2) current approaches adopted to address clients’ mental health needs; (3) future ideas for addressing clients’ mental health needs in the sexual health clinic; (4) definitions of, interpretations of, and reactions to syndemic theory. Parts 1–3 are described in a previous publication [2]; part 4 is the focus of the present article. Specifically, the interviewer asked the participant, “Have you heard of the term syndemic?” If they answered ‘yes’, the interviewer asked the participant to define the term; if ‘no’, the interviewer offered the following definition, adapted from Singer et al. [3]: “The word syndemic is a combination of the words synergy and epidemic. Syndemics happen when some kind of negative biological or behavioural problem exacerbates the negative health effects of two or more diseases or health conditions. It can involve negative interactions of diseases of all types like infections, mental health, non-communicable diseases, etc. Syndemics usually happen in populations that experience social inequalities.” All participants were then asked how the notion of syndemics affects their clinical practice. Interviews were audio-recorded and transcribed verbatim, and transcripts were subsequently checked for accuracy and anonymized.

### Analysis

Analysis was driven by the following questions: *How did participants describe syndemics? Which elements of syndemic theory were most salient to them and why? How and why did they embrace or resist the application of the theory to their work? And what, if anything, does the concept of syndemic add to their work, or the meanings they make of their work?* Our ultimate goal was to generate results that have the potential for application to clinical practice, specifically for sexual health clinicians [18].

Analysis began immediately following each interview; the first two authors exchanged impressions, questions, and tentative analytic ideas throughout data collection. We immersed ourselves in the data, reading and rereading the transcript, and for the present analysis, focused on part 4 of the transcripts [19]. Codes were applied at the level of statement (rather than word-by-word or line-by-line coding), and we examined each transcript and wrote participant-level analytic reflections, as recommended by interpretive description scholars [17, 18]. Early analytic assumptions and ideas were checked by presenting results to groups of sexual health providers (member checking) and asking for their reactions and interpretations. To increase the rigor of analyses, a third analyst (third author) read the excerpted part 4 from all transcripts and separately generated responses to the analytic questions outlined above. We then verified our initial, tentative findings with those generated by the third analyst for similarities and differences, and where necessary, adjusted our first impressions.

## Results

Our findings are presented in three parts. First, we interpret providers’ baseline knowledge and understanding of the notion of syndemics. Second, we answer the question, *how do syndemics create moral distress for sexual health providers?* Finally, we present findings from interviewees who began to imagine how we might prevent syndemic-related moral distress in sexual health settings.

### The term itself is academic: Providers’ baseline knowledge of syndemic theory

Responses to syndemic theory ranged widely. Some participants provided nuanced and clinically informed definitions; others expressed a vague familiarity with the theory but were unwilling to offer a definition; and others still had no prior knowledge of the theory. Participants who were previously aware of syndemic theory generally had more exposure to GBMSM clients, i.e., syndemic-burdened populations. For example, one nurse who works at an GBMSM-focused clinic offered the succinct definition: “*It’s basically in relation to gay men… different factors kind of compounding on one another to create higher [STBBI] risk*.” This nurse went on to explain the role of social stigma as a root cause to this syndemic: “*I think there’s a lot of pressure for people to fit into a heteronormative society because that’s how we’re all socialized from the beginning, whether or not we’re aware of it… and so I think the people who I see kind of have an understanding of that… of syndemics.*”

Although our interviews were framed explicitly in relation to mental health, when providers were presented with the concept of syndemic, many of them focused on syndemics involving two or more STBBI. As one nurse said, “*if you’re a gay man, and you have a rectal STI and you have syphilis, your rates, your chances of getting HIV are incredibly high compared to say a woman in that situation.*” In other cases, participants recognized that co-occurring conditions—in particular, substance use-related issues—increased STBBI risk but did not comment on the components of syndemic theory that point to social causes of syndemic conditions like stigma and exclusion in the case of GBMSM. One nurse remarked: “*We’re seeing a lot of cases of HIV and syphilis being diagnosed at the same time often even other diseases at the same time. High-risk men that have sex with men are frequently involved, and yeah drugs are often part of it as well*.”

Participants whose clinical shifts included outreach (or ‘street’) nursing—serving clients of diverse, but predominantly heterosexual orientations—were generally less familiar with the notion of syndemic, but readily identified the co-occurrence and interaction of multiple health conditions and the social production of syndemics in the client populations they serve. One outreach nurse promptly connected various components of syndemic theory when asked if the theory was relevant to their work:

*Most of the people I work with are caught in several big traps, right? Poverty, addiction, mental illness, which to me […] I believe a lot of mental illness comes out of a broken social system, not from an organic, you know, brain dysfunction. I think, you know, in an ideal world, everyone would be included somehow, right? […] there would be a way for each individual to get what they needed […] their basic needs met including needs for socialization, and needs for love, and a community, and all of that, right?*

Once participants were provided with a definition of syndemic, they consistently affirmed the importance of the theory in explaining population-level phenomena, but occasionally resisted its relevance for clinicians—who treat individuals, not populations. One public health nurse questioned how a theoretical and epidemiological concept translates to treating individuals:

*Interviewee: Well, I mean the term itself is academic. When you give the definition, I’m like, “ok, two or more illnesses that become linked, or they affect the presentation of one another, I guess.”*

*Interviewer: And how would you use that concept in your daily practice?*

*Interviewee: It doesn’t really connect in terms of how I see patients on a day-to-day basis, and how we kind of relate to them and treat them […] the term makes a bit more sense on the population level.*

While few participants connected all of the components of syndemic theory (social causes + co-occurrence of health conditions + interaction/exacerbation of health outcomes), many participants expressed ways in which individual components of the model resonated. In particular, providers consistently reflected that they observe multiple co-occurring mental health needs in their clients, especially anxiety:

*I would be a really good STI nurse if I was a really good psychologist because you know there’s so much anxiety that comes in with a lot of situations. Anxiety about “do I have something?” And the phone calls […] Those phone calls are a lot about anxiety about having something and rechecking and rechecking […] Someone has done some behavior that’s not their norm and they’re having a hard time letting go of whatever that was because it was something that they don’t normally do. And now they’re really freaked out that there’s going to be such a big negative consequence from it.*

Several participants asked whether syndemic theory was the same as intersectionality, another theoretical “lens” which has grown in popularity and which also incorporate concepts of social production of health and interactions between factors—though in the case of intersectionality frameworks, the interacting factors are the social positions (i.e. sexual identity, gender, race, class) and the forms of oppressions faced (i.e. homophobia, sexism, racism, classism), rather than health outcomes [20, 21]: *“[Interviewer: Have you ever heard of that term before?]… [Nurse participant:] Yeah, I guess, is ‘intersectionality’, is that related to or is that different from ‘syndemic’?*” One nurse suggested that the concept of ‘intersections’ made more sense when discussing relationships between sexual and mental health (the framing of our study): “*I think it [syndemics] totally makes sense. It’s just interesting to see that they have a term for it, because nothing happens in isolation*.” Another nurse connected both syndemic and intersectionality frameworks to Rhodes’s risk environments framework, which shifts attention away from an individual “at-risk” by virtue of behaviors and toward a social, physical, and political environment which includes resources that may structurally reduce “risk” of outcomes, especially HIV and STBBI [22]:

*Interviewer: Have you heard the term ‘syndemic’ before?…*

*Interviewee: I think this guy Rhodes has this terminology for the ‘risk environment’, and it’s sort of about moving interventions from being focused at the individual to being focused at the community […] like I couldn’t believe that they admitted and put that on paper, I was like “this should change the world!” because we’re actually saying that these are the greatest factors […] and then if you add to it, that they interact together […] I think it’s really important when we actually quantify oppression and like social things, we need to have that. That’s why intersectionalism [sic] is so good in that way, you know when we’re talking about it at the next level.*

All three frameworks—syndemic, intersectionality, and risk environments—posit social structures as the root cause of health problems. Participants in this study repeatedly reflected that the ‘downstream’ health problems—HIV or other STBBI, in the case of their particular contexts—are not treated until there is a diagnosable condition. Thus, on the one hand, participants rejected these theories as being too “academic,” and therefore less relevant to their day-to-day work; on the other hand, when further prompted, clinicians affirmed the relevance of these theoretical frameworks in drawing attention ‘upstream’, to the environmental socio-structural factors that continue to concentrate STBBI, and correlated mental health concerns, in the same groups of patients.

### You can go too deep: Moral distress in the context of syndemics

Moral distress manifested in the practices of clinicians when they had to strike a balance between probing to gather more context—a practice that would sensibly follow from a syndemic-oriented approach to care—and probing too deeply, thereby opening a set of concerns that were out-of-scope for a sexual health encounter. Clinicians often described finding themselves in a double bind, wherein they felt compelled to ask broader questions that would explore the psychosocial circumstances that brought their client to the clinic, but then felt unprepared to address those broader psychosocial needs. Interviewees repeatedly shared holistic assessment strategies, like this physician:

*[I] try to ask more questions about what this person’s life is like, what’s a day to day like? […] ‘Okay, tell me about what brought you here today. What’s your life like at home? Where do you, you know, what’s your living situation? What’s your work situation like? […] Do you feel safe? And I think that’s helpful even in STI, like you think about relationships, do you feel safe? Because I think all of that stuff affects, you know, the choices people make in terms of their risk behaviours […] and it also gives me a—I think it helps me be a little bit more empathetic, right?*

These same clinicians reflected how they had to learn—through practice (rather than explicit training)—how to set limits on these strategies; as one nurse described:

*I think we, well I, operate more as a compassionate listener. Because I don’t have, I don’t have a background in mental health and I don’t have counselling skills really, so I don’t know how much good it really does, but at least someone knows they can come and talk to somebody. I can’t, like you know, I don’t have strategies to offer people that will really make a big impact in their mental health.*

In one interview, a nurse listed a series of social, mental health, and other health needs, beyond STBBI prevention and treatment, describing clients who were “*homeless, underhoused, [with] polysubstance use, alcohol, smokers, all these coinfections*…”, prompting the interviewer to ask, “*I just can’t help but wonder then, like what do you do with that? Because, first of all, that’s a lot for you to take on as a provider*.” They replied:

*Oh yes… you can go too deep, and then you’ve left them [the patient] unsettled because you’ve opened a wound that they can’t deal with right now, and we're not providing support for them to deal with it right now, and that’s totally unfair to that client. You can’t do that to someone and let them walk out like that. So, I'll just kind of gauge where they’re at and see how far you can go without causing further harm but trying to support what’s happening in front of you. But it is a balancing act, always, and everyone is so different, everybody's so different and it’s incredibly challenging work.*

Here, moral distress stemmed from a dilemma between their tendency to elicit their clients’ histories of trauma—to engage more deeply with the client—but then encountering the risk of retraumatizing or distressing the client without the possibility of offering tangible solutions to their need. The “balancing act” described by this provider requires striving to provide optimal care for those patients experiencing syndemics, while limiting the risk of opening a set of concerns that is beyond the provider’s or clinic’s scope of practice.

Some interviewees were able to go a step further to identify structural (i.e., systemic healthcare) causes to syndemic-related moral distress. They described ways in which the STBBI mandate of their workplace created a particular form of distress, when they saw non-STBBI healthcare needs of clients but felt that addressing these needs took them outside of their employer’s mandate. One clinic coordinator (nurse) explained:

*Sometimes I feel really sad when I walk away from those [clients’ stories of physical abuse] situations, because I don't know what is going to happen. And there is only so much we can really take on. Like I have 20, maybe 25 minutes to see them, and really our mandate is sexual health, but it's tricky, because we can say, “only sexual health,” but there’s a lot more to it.*

A street outreach nurse working with populations experiencing insecure housing, high rates of injection substance use, and multiple barriers to gainful employment cited their program’s explicit expectation related to HIV testing and diagnosis volume. They reflected on the tension this creates when trying to address their clients’ other health-related needs. They described these needs as wide-ranging, for example, requiring first aid and medication, or help with paperwork to apply for housing. As complex as these needs are, the nurse explained that they are all prerequisites to uptake of HIV-focused public health interventions, like testing, prophylactic medication, and treatment. Therein, lies the moral distress; in their words:

*So, [we’re] working under the communicable disease [CD] umbrella, so basically our mandate, as you probably know, is STI and CD treatment and testing. Immunizations and things like that. And to engage people into that care we have a few things that we, that we offer – over-the-counter medications and doing wound care. Wound care and vein maintenance and things like that as well for people who use [substances]. So, it’s, it’s a pretty big coup in the day if we can get someone to test just based on the peoples’ lives with chaos wherever we’re located… that’s our mandate but it happens so rarely. Like yesterday, I tested one person out of how many people? I saw 30 people that day […] then I think, I’m not doing what I’m—my purpose of this program is. […] That can be really, really tough.*

For this provider, their clients’ healthcare priorities did not always align with those of the HIV-focused funding mandate of their program. These reflections demonstrate how the source of syndemic-related moral distress is not only the social contexts of patients’ lives but also how the HIV and public health services themselves are organized—often established to respond to a single health issue at a time.

### People are understanding more the value in partnerships: strategies to prevent or manage syndemic moral distress

In this final section, we turn to the question of what it will take to address the phenomenon of syndemic moral distress. Most participants were hard-pressed to identify actionable interventions that could relieve the distress they described when responding to syndemics. As one counselor lamented, “*The problem is that… ‘syndemic’ does a really good job of explaining a problem but lacks the research and underpinning to similarly describe a solution*.” This participant succinctly summed up what many interviewees expressed, a doubt that syndemic theory could point to an intervention at the level of a sexual health clinic. While most participants indicated that ensuring an *inclusive* environment for syndemic-burdened populations was critical at the sexual health clinic, addressing upstream causes of syndemic were simply out of scope. A clinic coordinator (nurse) summed up this perspective as follows:

*Interviewer: Does a syndemic approach, or this way of thinking of healthcare, does it have traction in sexual health?*

*Interviewee: You are thinking about ‘why is this community experiencing this increased incidence of these things […] it’s complicated, and I think there is stuff that we can do here in terms of harm reduction and inclusivity and access to say, non-judgmental services, but then there is obviously stuff that needs to change at a systemic level […]like I think we are at kind of the tip of the iceberg here. Like we are doing what we can, but there is so much else that we can’t always address.”*

In the context of these limitations, two actionable strategies emerged: (1) partnering with non-health-related organizations to improve the *upstream* social conditions of syndemic-burdened populations (i.e., before they get to the sexual health clinic), and (2) syndemic-related screening as a method to limit or focus attention on particular clients, thereby minimizing the moral distress that may occur when a provider “goes too deep” (after clients get to the clinic). With regard to the upstream solution, one nurse reflected on their long career in HIV and sexual health clinical work, noting a shift over time, with increasing reliance upon community partners, especially those offering psychosocial support services:

*I really feel like earlier on, just as far as the training, it was very focused on the STI, so like sexual health assessment specifically and then the testing procedures and then sort of then focusing on the outcomes, so results that come up and the counselling that happens around that and referrals that happen but specific to STI… Whereas I feel like now it’s a lot broader. I think people are understanding more the value in partnerships like [names of local lesbian, gay, bisexual, queer, and transgender community centres], where it’s a centre for the community and there’s resources that are offered in the centre, of which STI is just one. So just kind of physically it’s like the space is different. It’s not quite so STI central. It’s more…I feel like it’s more holistic.*

Other interviewees similarly offered practical ways for more holistic health promotion services to be supported by sexual health clinics through coalitions and collaborations. Providers variously spoke of how they operated services—outreach or clinics—in collaboration with high schools, community centres, immigrant and refugee groups, complementary health clinics, sex worker resource centres, transitional housing services, harm reduction programs, addictions treatment facilities, and sexual assault services, among others. As one clinic coordinator (nurse) explained, “*the way we’re expanding services is through partnerships. Right, so things like [name of a youth clinic], or, you know, occasional other high school clinics where the [public] health authority pays for part, or whatever. Any partnership is how we’ve been able to expand*.”

Other clinicians pointed to downstream solutions that involved targeted syndemic-informed screening. As one nurse suggested, “*we should be asking specific questions routinely to help sort of screen, and try and capture those clients that are struggling, and have unmet mental health or addiction needs*.” Several participants indicated that they would be more likely to offer a mental health referral to a client affected by syndemic-related processes, particularly heterosexism and anti-gay stigma. One nurse reflected, “*I mean, we all have mental health stuff, but like the population I work with [specifically referencing gay men] is more likely to have mental health concerns.*” In this instance, the clinician agreed that, in the context of syndemics, sexual orientation may be a reasonable variable to be used when prioritizing the administration of mental health-related screening tools in a sexual health clinic.

## Discussion

Our interviews with 22 sexual health providers in British Columbia, Canada suggest that although knowledge of syndemic theory and perceived applicability of syndemic theory to sexual clinical work are both limited, the high prevalence of syndemic-burdened clients in their practice created distress. We define ‘syndemic moral distress’ as a moral impulse of sexual health providers to address clients’ psychosocial health needs related to STBBI and attributable to syndemic conditions, which is limited by the following factors, thus creating distress for the provider themselves. Factors identified by our participants that constrain their individual-level responses to syndemic conditions and create moral distress include limited specialized training in mental health, a risk of leaving patients ‘unsettled’ from probing beyond immediate STBBI concerns, and the clinic’s mandate to deliver STBBI-specific care and treatment.

Clinicians generally concern themselves with how best to treat *individual patients*, whereas epidemiologists (and other population scientists) generally concern ourselves with understanding how to treat *populations* [23]. Given this difference in disciplinary purviews, it is perhaps not surprising that clinicians participating in this study at times resisted an application of syndemic theory—which was born out of population-level anthropological and epidemiological research [3]—to their clinical practices. Despite this reluctance to describe their practices in the language of syndemics, many of the clinicians we interviewed saw the value of the syndemic concept, insofar as it validated the importance of upstream psychosocial stressors on the lives of their patients. For this reason, we believe that syndemic theory does have relevance to sexual health clinicians, so long as their experiences of moral distress are acknowledged and addressed. Some participants invoked intersectionality or risk environment frameworks to draw cogent connections between their clients’ social environments and more immediate health-related needs. This suggests that sexual health clinicians are attuned to upstream contributors to syndemics, whether they conceive of them within a syndemic framework or some other ecological model. This sensitivity to structural factors may be useful in bolstering support to some of the upstream interventions suggested below. Clinicians experience moral distress while responding to their patients’ complex interactions between social circumstances, psychological and substance use struggles, and STBBI diagnoses and related concerns. We foresee practical implications of this research both within and beyond the sexual health clinic. Preventive approaches to syndemic moral distress have yet to be described in detail, but we tentatively suggest that these would be consistent with broader calls for actionable, syndemic-informed interventions at the clinic level [3, 8, 24]. More specifically we suggest: (1) tailored syndemic-informed screening for unmet mental health needs, in order to focus clinicians’ time and energy and thereby reduce syndemic moral distress; (2) educational and dialogical interventions that normalize syndemic moral distress and allow providers to self-reflect, analyze, and communicate with clinical teammates when experiencing syndemic moral distress; and (3) community partnerships and structural reforms to reduce syndemic-related burdens *upstream*, i.e., before clients arrive at the sexual health clinic.

Participants of this study have suggested tailored screening for mental health services within sexual health service settings, prioritizing members of populations most affected by syndemics. This strategy may reduce moral distress for sexual health providers by focusing their energy on clients with the greatest need for psychosocial supports. In response to this suggestion, our team has developed an online database of low-barrier, low-cost, and lesbian, gay, bisexual, queer, transgender, and Two-Spirit-affirming mental health and substance use services, which is now being used by sexual health clinicians across the province of British Columbia (MindMapBC.ca). Syndemic-related screening has been trialed elsewhere in North America, Australia, and Europe, with early results suggesting acceptability and potential benefits—though larger long-term studies are needed [25–28].

We further suggest that sexual health clinicians may benefit from interventions that have been used in other healthcare settings to manage moral distress. Such interventions are typically participatory—including administrators and clinicians occupying a variety of roles within the clinic—and facilitated by health ethicists, who offer structured sessions to normalize and debrief experiences of moral distress, with opportunities to propose and refine clinic and health systems policies to manage distress [15]. Other individual-oriented interventions include encouraging self-reflection during moments of moral distress, which may help providers to detect and describe patterns of syndemic moral distress, as they occur encounter-to-encounter [29].

An overemphasis on biomedical solutions is unlikely to ultimately resolve syndemics, given that syndemics are rooted in social and structural causes. Therefore, we recommend structural changes beyond these individual clinician-focused interventions, to reduce the ‘siloing’ of STBBI, mental health, and substance use services, and in turn to the syndemic moral distress described in our study. Structural changes may include bundling and co-location of services and expansion of low-barrier mental health management services—acknowledging that in Canada, most mental health services are outside of the publicly funded service bucket, available only to those who are fortunate enough to have expendable income or private health insurance, e.g., through an employer [30]. We are heartened by providers who spoke of how they operated STBBI services in collaboration with high schools, community centres, transitional housing services, harm reduction programs, addictions treatment facilities, sexual assault services, and many others, and we foresee potential improvements in population health outcomes as more of these services are integrated, or at least supported through partnerships with sexual health clinics.

While this study introduces the relevance of syndemic theory (and other ecological models) to the work of sexual health practitioners, it leaves many other questions unanswered. We did not systematically collect detailed characteristics about participants, such as age, gender, levels of training, years of experience—a limitation which should be remedied in future studies of this nature. The majority of participants were nurses, which limited our ability to make comparisons across the professions. Finally, we recognize that the perspectives shared here are those of providers, which must be complemented by research with sexual health service users.

To arrive at the innovations in clinic organization and health service delivery outlined above, we propose the following action research initiatives, which may help address some of the limits of our exploratory qualitative study presented here. As a next step, we will conduct in-depth consultations with patients to understand the perceived benefits and drawbacks to syndemic-informed service bundling or integration from their perspectives. Second, we propose health economics research which can help demonstrate to policymakers the system-level cost savings of increasing access of early, preventative mental health care to this patient population. Third, implementation research is needed to examine how best to implement and scale some of the ideas generated through our research, within the sexual health clinic setting—including tailored syndemic-informed mental health screening, structured approaches to normalizing and managing syndemic moral distress, service bundling/integration, and community partnerships.

## Data Availability

The datasets generated and analysed during the current study are not publicly available due to the sensitive nature of the interviews and privacy protections required by the Research Ethics Board; however, non-identifying data are available from the corresponding author on reasonable request.

## Abbreviations

BC: British Columbia
GBMSM: Gay, bisexual, and other men who have sex with men
STBBI: Sexually transmitted and blood-borne infections

## Footnotes

1 We use the terms patient and client interchangeably throughout this manuscript, given that the providers we interviewed variably described the individuals they serve as “patients” or “clients,” despite working in the same clinics, with the same patients/clients.
